# Expanding contraceptive choice among first-time mothers age 15–24 in Kinshasa: The Momentum pilot project

**DOI:** 10.3389/fgwh.2023.1087009

**Published:** 2023-02-13

**Authors:** Anastasia J. Gage, Francine Eva Wood, Rianne Gay

**Affiliations:** ^1^ Department of International Health and Sustainable Development, Tulane University, New Orleans, LA, United States; ^2^ Center on Gender Equity and Health, Department of Medicine, University of California, San Diego, La Jolla, CA, United States; ^3^ Tulane International, LLC, Kinshasa, Democratic Republic of the Congo

**Keywords:** informed choice, contraceptive use, first-time mothers, long-acting reversible contraception, youth, Democratic Republic of the Congo (DRC)

## Abstract

**Introduction:**

Evidence shows that an expanded range of contraceptive methods, client-centered comprehensive counseling, and voluntary informed choice are key components of successful family planning programs. This study assessed the effect of the Momentum project on contraceptive choice among first-time mothers (FTMs) age 15–24 who were six-months pregnant at baseline in Kinshasa, Democratic Republic of the Congo, and socioeconomic determinants of the use of long-acting reversible contraception (LARC).

**Methods:**

The study employed a quasi-experimental design, with three intervention health zones and three comparison health zones. Trained nursing students followed FTMs for 16 months and conducted monthly group education sessions and home visits consisting of counseling and provision of a range of contraceptive methods and referrals. Data were collected in 2018 and 2020 through interviewer-administered questionnaires. The effect of the project on contraceptive choice was estimated using intention-to-treat and dose-response analyses, with inverse probability weighting among 761 modern contraceptive users. Logistic regression analysis was used to examine predictors of LARC use.

**Results:**

Project effect was detected on receipt of family planning counseling, obtaining the current contraceptive method from a community-based health worker, informed choice, and current use of implants vs. other modern methods. There were significant dose-response associations of the level of exposure to Momentum interventions and the number of home visits with four of five outcomes. Positive predictors of LARC use included exposure to Momentum interventions, receipt of prenatal counseling on both birth spacing and family planning (age 15–19), and knowledge of LARCs (age 20–24). The FTM's perceived ability to ask her husband/male partner to use a condom was a negative predictor of LARC use.

**Discussion:**

Given limited resources, expanding community-based contraceptive counseling and distribution through trained nursing students may expand family planning access and informed choice among first-time mothers.

## Introduction

In high-fertility sub-Saharan African countries, it is estimated that one in four adolescent girls and young women want to plan and space their pregnancies but are not using a method of contraception. However, unmet need for contraception is not uniform across these countries and ranges from 18% in Niger to 43% in Angola (1). Providing a wide range of modern contraceptive methods, whether through health facilities or community-based distributors, as well as client-centered comprehensive counseling enable women and couples to make informed and voluntary decisions about family planning (FP) use (2). These factors and a constant supply of affordable contraceptive methods contribute to increased and continuous use of modern contraceptives.

In Democratic Republic of the Congo (DRC), the modern contraceptive prevalence rate among currently married women increased from 8% in 2007 (3) to 18% in 2017–2018 (4). Nationwide, few currently married adolescent girls and young women were using a modern contraceptive method (age 15–19: 10%; age 20–24: 15%). The modern contraceptive prevalence rate was higher among sexually active unmarried adolescent girls and young women (19% among 15–19-year-olds and 30% among 20–24-year-olds) (4). The capital city, Kinshasa, was the only area of the country to meet the 2020 modern contraceptive use target of 19% set by the National Strategic Plan for Family Planning (5). The prevalence of modern contraceptive use among married women in the city increased from 19% in 2013 to 30% between 2020 and 2022 (6), but was low compared to other large cities in sub-Saharan Africa. Unmet need for contraception has remained high and in 2018 was higher among currently married adolescents aged 15–17 (44%) than among those aged 18–19 and 20–24 (30% and 32%, respectively) (4). More than half of all unmarried Congolese women age 15–19 had an unmet need for FP, with the estimate as high as 66% among 15–17-year-olds (4). Most adolescent pregnancies in Kinshasa were unintended (80% in the age group 15–19) and almost half of those pregnancies ended in abortion (49%) (7).

Expanding access of to a full range of contraceptive methods is a critical first step in addressing the dual challenge of high unmet need and high unintended pregnancy. With the rapid increase in the use of long-acting reversible contraception [LARC, i.e., subdermal contraceptive implant and intrauterine device (IUD)] in the DRC (8) and other African countries (9), studies have sought to understand the sociodemographic and attitudinal factors underlying the choice between no method, traditional methods, short-term methods, and LARCs. Findings have indicated that older age and being currently married are significant sociodemographic determinants of women's choice of LARC (2). Positive predictors of current use of the implant and intention to use it in the future have included knowledge about the safety of the implant, beliefs about its effectiveness, the ease of insertion and removal, and support from intimate partners (10). Other predictors of LARC use have included history of spontaneous or induced abortion, desire to limit family size, accurate knowledge of LARC, less frequent medical visits, health professional advice, and high economic status (11); as well as race/ethnicity, relationship status, and school type (12). History of delivery and induced abortion were strongly associated with choosing LARC, especially among women less than 25 years of age (13), as were high IUD knowledge and earlier onset of sexual activity among unmarried young adults (14).

Focusing on contraceptive continuation and clients' rights to informed contraceptive choice, some studies have assessed counseling content by measuring clients' receipt of information about (a) other methods aside from their current method, (b) possible side effects from their current method, and (c) what to do if they experienced side effects. The Method Information Index (MII), calculated as an affirmative response to all three issues, measures the extent to which adequate information is provided to help women make informed decisions about contraceptive use (15). A comparative analysis of the MII using data from the Demographic and Health Surveys suggested that the quality of contraceptive counseling varied significantly across the 25 countries examined and by method, household wealth, and education within countries (16). In some instances, researchers have gone beyond this proxy indicator of counseling quality to assess a client's ability to obtain her method of choice and whether the client or someone else was primarily responsible for deciding whether to use FP and which method to use. Receipt of information on side-effects of the selected method and facility readiness to provide a range of contraceptive methods were significantly associated with receipt of the method choice in rural India (17).

Although adolescent girls and young women face unique barriers to contraceptive use, including lack of confidentiality, cost, limited access to sexual and reproductive information and services (18), and lack of autonomy in contraceptive decision making (18–20), few studies have examined full, free, and informed choice among youth. The objectives of the present study were to (a) estimate the effects of the Momentum project on community-based sources of contraceptive supply and contraceptive choice among first-time mothers (FTMs) age 15–24 at baseline who were currently using modern contraceptives at endline, (b) determine the extent to which the project's effect on these outcomes varied across sociodemographic groups; and (c) identify sociodemographic factors that influenced adolescent and young contraceptive users' choice between LARCs and short-term methods.

The study is of great programmatic relevance in view of the high adolescent childbearing and unintended pregnancy rates in the DRC. The study contributes to the literature by broadening understanding of the extent to which interventions have been effective in increasing informed and voluntary choice of contraceptive methods and access to LARCs among urban adolescent girls and young women. As LARCs are not coital- or user-dependent, they are more effective than short-term methods in preventing unintended pregnancy among subgroups of young women who are at high risk of inconsistent contraceptive use. Hopefully, knowledge gained from our study will help programs better address FTMs' contraceptive needs in Kinshasa.

## Materials and methods

### Study design

Momentum was an integrated FP and maternal and newborn health project. The project sought to increase contraceptive uptake and the adoption of health-seeking behaviors and household practices beneficial to mother and baby, and foster gender-equitable attitudes and behaviors among FTMs age 15–24 years and their male partners in Kinshasa, DRC. The study design was quasi-experimental, with three intervention and three comparison health zones. FTMs were recruited through convenience sampling at high-volume maternal health facilities and community sites and were followed up for 16 months. Enrollment criteria were: (a) six-months pregnant with the first child; (b) willing and mentally competent to provide informed consent; (c) ability to speak French or Lingala; and (d) residence in the intervention or comparison health zones. A total of 1,927 FTMs were completely interviewed in both surveys, of whom 761 were currently using a modern contraceptive.

In intervention health zones, trained nursing students conducted monthly group education sessions and home visits that included client-centered counseling on postpartum FP and birth spacing, offered a range of contraceptive methods (Implanon NXT, Sayana® Press, progestin-only pills, combined oral contraceptive pills, male condoms, emergency contraception, and Cycle Beads), and provided referrals. Group education sessions were based on Program M (21), a curriculum that focuses on equitable gender roles, empowerment in interpersonal relationships, and sexual and reproductive health and rights. Each FTM was assigned a dedicated pair of trained nursing students (one male and one female) who conducted monthly home visits in the prenatal and postnatal periods.

Data were collected through face-to-face interviews conducted by trained data collectors at baseline (September to November 2019) and endline (May to July 2020). Pretested questionnaires were used to gather data on the following FP-related topics: background characteristics; knowledge of, attitudes toward, and use of contraceptive methods, perceived norms, informed choice, decision making about contraceptive use, method satisfaction, and exposure to Momentum interventions. Questionnaires were administered using Open Data Kit software and Android smartphones.

### Variables

Six binary outcome variables were measured: (a) receipt of FP counseling from a community-based health worker who visited the household in the past 12 months; (b) obtaining the current contraceptive method from a community-based health worker (defined to include Momentum nursing students who were community-based distributors); (c) informed choice (i.e., whether the provider informed the FTM about all of the following: other FP methods that she could use, possible side effects or problems that she might have with the method, and what to do if she experienced any side effects or problems, i.e., the MII); (d) current use of implants; (e) current use of injectables; and (f) current use of LARCs vs. short-term modern contraceptive methods (female and male condoms, injectables, pills, Cycle Beads, and emergency contraception). As the analysis was based on users of modern contraceptive methods, women who reported not doing anything to prevent pregnancy and those using traditional methods (withdrawal and rhythm) were not included in the analysis. Secondary outcomes included the FTM's participation in decision making about her current contraceptive method and method satisfaction.

All treatment effect models controlled for baseline measures of age, marital status, years of schooling, ethnicity, parents' education, and weekly television viewing. For dose response, we used multiple measures of intervention exposure: level of exposure categorized as full (participation in both home visits and group education), partial (one of the two) and no exposure (neither); the number of home visits (none, 1–3, 4–6, 7+), the number of group education sessions (none, 1–2, 3–4, 5+), and the total number of exposures to Momentum, defined as the number of home visits plus the number of group education sessions (none, 1–3, 4–6, 7–9, and 10+).

Like the treatment effect models, the multivariable regression model of the choice of LARCs over short-term methods was restricted to women who were currently using a modern contraceptive method at the time of the endline survey. The regression controlled for level of exposure to Momentum interventions (none (comprising users in the comparison health zones as well as 45 users in the intervention health zones who were not exposed to any Momentum interventions), partial (either home visits or group education sessions), and full (both home visits and group education sessions)); receipt of counseling on FP and/or birth spacing during the prenatal period, which was measured at baseline and consisted of the following categories: none, FP or birth spacing, and both FP and birth spacing); being never married at baseline (yes vs. no); Bakongo ethnicity (yes vs. no); worked in the past 12 months at baseline (yes vs. no); awareness of LARCs (a binary variable indicating that the respondent had ever heard of IUDs and implants); and household wealth at baseline [low (reference group), medium, and high]. We also controlled for the FTM's perceived ability to say “no” to unwanted sex (yes vs. no) and to ask her husband/partner to use a condom if she wanted him to (yes vs. no); whether the pregnancy was unintended at baseline (yes vs. no); and age group (15–19 vs. 20–24).

### Statistical methods

Descriptive statistics were used to summarize participants' characteristics, sources of supply, and outcomes of interest. To estimate the effects of the Momentum interventions, we conducted intention-to-treat analyses (intervention health zone vs. comparison health zone) and dose-response analyses (for level of exposure to Momentum) using treatment effects models with inverse probability weighting. Results were presented as average treatment effects (ATE) and average treatment effects on the treated (ATET). The ATE is the effect that would have been observed had the entire sample of current users of modern contraception been treated. The ATET is the effect among those who participated in the Momentum interventions. We conducted a balance analysis for the treatment effects. The overidentification test for covariate balance yielded a *p*-value of 0.507, signifying that the model-adjusted means of the covariates were the same between the intervention group and the comparison group. The analysis of the effect of Momentum interventions was disaggregated by selected sociodemographic variables (e.g., age group, marital status, and household wealth) to obtain a picture of possible social inequities in Momentum's effect on contraceptive choice.

Logistic regression analysis was used to determine predictors of current use of LARCs vs. short-term methods and included a variable for treatment effect. Results of the analysis were presented in the form of adjusted odds ratios (aOR) with 95% confidence intervals (CI). There were 761 current users of modern contraceptive methods. As questions on the FTM's ability to say no to unwanted sex and request that her husband/partner use a condom were restricted to women who were currently married, living together, or had a romantic partner, the sample for the regression model for LARC use was further restricted and was based on 699 FTMs. The variance inflation factor for the regression analysis was 1.15, which suggested that multicollinearity was not of concern. As indicated by Supplementary Table S1, compared to their counterparts with non-missing values, significantly more modern contraceptive users with missing values were never married (48% vs. 28%) and declared their pregnancy as unintended (93% vs. 83%). These differences should be considered in interpreting the results.

## Results

### Sample characteristics

Of the 761 FTMs who were currently using a modern method of contraception, just over half (415/761) lived in the intervention health zones (see Table 1). Two in five FTMs had completed secondary school or higher levels of schooling, one in four were never married, a third were employed, almost two-thirds watched television weekly, and four in five had two parents with secondary or higher education. The only significant difference between intervention and comparison health zones was in the prevalence of LARC use, which was significantly higher in intervention than in comparison health zones (40% vs. 23%). In intervention health zones, 62% of modern contraceptive users participated in both home visits and group education sessions, 11% had no contact with the project, and 27% had 10 or more contacts. Non-participation in group education sessions was three times as high as non-participation in home visits (35% vs. 11%).

**Table 1:** Percent distribution of first-time mothers age 15–24 who were currently using a modern method of contraception by baseline characteristics and health zone, Kinshasa.

Characteristics	Health Zone				Total	
	Comparison		Intervention			
	%	No.	%	No.	%	No.
**Age group**						
15–19	45.1	156	49.6	206	47.6	362
20–24	54.9	190	50.4	209	52.4	399
**Highest level of schooling**						
None/prim/sec. inc.	56.1	194	58.8	244	57.6	438
Sec. complete/higher	43.9	152	41.2	171	42.4	323
**Marital status**						
Ever married/formally engaged	71.7	248	71.3	296	71.5	544
Never married	28.3	98	28.7	119	28.5	217
**Household wealth**						
Low	32.7	113	33.5	139	33.1	252
Medium	31.5	109	36.4	151	34.2	260
High	35.8	124	30.1	125	32.7	249
**Worked last year**						
No	59.8	207	66.5	276	63.5	483
Yes	40.2	139	33.5	139	36.5	278
**Watched TV weekly**						
No	35.0	121	36.1	150	35.6	271
Yes	65.0	225	63.9	265	64.4	490
**Both parents have secondary/higher education**						
No	20.2	70	20.5	85	20.4	155
Yes	79.8	276	79.5	330	79.6	606
**Method type** ***						
Short-term method	77.5	268	60.0	249	67.9	517
LARC	22.5	78	40.0	166	32.1	244
**Source of supply for current method** ***						
Public sector	21.7	75	20.5	85	21.0	160
Private sector	69.6	241	43.4	180	55.3	421
Other source	8.7	30	36.1	150	23.7	180
**Level of exposure to Momentum**						
None			10.8	45		
Partial			27.5	114		
Full			61.7	256		
**No. of home visits**						
0			14.9	62		
1–3			26.8	111		
4–6			34.2	142		
7+			24.1	100		
**No. of group education sessions**						
0			34.9	145		
1–2			1.5	6		
3–4			3.1	13		
5+			60.5	251		
**No. of times exposed to Momentum**						
0			10.8	45		
1–3			16.4	68		
4–6			26.5	110		
7–9			19.3	80		
10+			27.0	112		
Total	100.0	346	100.0	415	100.0	761

Source: Momentum 2018 Baseline Survey and 2020 Endline Survey.

*** *p* < 0.001 for health zone differences.

In both study arms, private medical institutions were the most common sources of contraceptive supply. However, significantly more current users of modern contraceptives obtained their method from private medical institutions in comparison health zones than in intervention health zones (70% vs. 43%). In both study arms, pharmacies were the most common private medical source for contraceptive methods (data not shown). Public institutions, most of which were government health centers, accounted for approximately 21% of contraceptive supply. Reliance on other sources of contraceptive methods was four times as prevalent in intervention health zones than in comparison health zones (36% and 9%, respectively; see Table 1) due largely to community-based provision of contraceptive methods by Momentum nursing students (30%; data available upon request).

### Bivariate results

Table 2 shows that there were significant differences between comparison and intervention health zones in the percentage of current users of modern contraceptives who received FP counseling from a community-based health worker who visited the household in the past 12 months (2% vs. 55%) and in the percentage who obtained their current method from a community-based health worker (4% vs. 33%). Differences by study arm were statistically significant (*p* < 0.001) for each sociodemographic subgroup (not shown) and outcome. In intervention health zones, at least two in five current users of LARCs (there was only one IUD user) obtained their method from a community-based health worker compared to one in four users of short-term methods (not shown).

**Table 2:** Percentage of modern contraceptive users age 15–24 with selected contraceptive outcomes, by age group and health zone, first-time mothers, Kinshasa.

	Age 15–19			Age 20–24			Total		
Contraceptive Outcome	Comparison	Intervention	Sig.	Comparison	Intervention	Sig.	Comparison	Intervention	Sig.
Received FP counseling from CHW	1.9	51.9	***	3.2	57.9	***	2.3	54.9	***
Obtained current method from CHW	3.2	29.6	***	5.3	36.8	***	4.3	33.3	***
**Provider informed FTM about:**									
Other contraceptive methods she could use	37.8	64.1	***	45.8	70.8	***	42.2	67.5	***
Side effects of the method	37.2	56.8	***	37.9	55.5	***	37.6	56.1	***
What to do about side effects	35.3	56.8	***	35.3	55.0	***	35.3	55.9	***
Method Information Index	30.8	43.2	*	30.0	45.5	***	30.3	44.3	***
Currently using implants	27.6	41.3	**	18.4	38.3	***	22.5	39.8	***
Currently using injectables	16.7	18.4		19.5	18.2		18.2	18.3	
*N*	156	206		190	209		346	415	

Source: Momentum 2018 Baseline Survey and 2020 Endline Survey.

CHW, community-based health worker.

Within comparison health zones, the age difference in the prevalence of implant use was statistically significant [Pearson chi2(1) = 4.101; *p* = 0.043].

*** *p* < 0.001; **p < 0.01; *p < 0.05. *P*-values pertain to differences between health zones.

More current modern contraceptive users in intervention health zones than in comparison health zones were informed about other methods that they could use (68% vs. 42%), side effects of the method used (56% vs. 38%), and what to do if they experienced side effects or problems with the method (56% vs. 35%). The data suggested that in both study arms, FP providers were somewhat more likely to inform users of modern methods about other methods that they could use than about potential side effects and what to do if they experienced side effects. It is worth noting that in both study arms, more LARC users were informed about of each component of the MII (81%-83%) compared to users of short-term methods (22%-57%) (data not shown).

Only 30% of current users of modern contraception in comparison health zones and 44% of those in intervention health zones reported receiving information on all three items (see Table 2). The MII ranged from 19% among users of short-term methods in comparison health zones to 71% among their counterparts who were LARC users (results available upon request). Health zone differences in the MII were statistically significant in all socioeconomic groups except those who were more educated, resided in the wealthiest households, and used LARC (not shown). Implant use was significantly more prevalent in intervention health zones than in comparison health zones (40% vs. 23%) in the total sample of current users of modern contraceptives and in all socioeconomic groups examined (not shown). The percentage of current users of modern contraceptives who used injectables vs. other modern methods was 18% in both study arms.

As shown in Supplementary Table S2, about 80% of FTMs chose their current contraceptive method either alone or jointly with their husband/partner. Health zone differences in this outcome were not statistically significant in any sociodemographic sub-group. The prevalence of method satisfaction, defined as the percentage of modern contraceptive users who reported that they were very satisfied with their current method, was significantly higher in intervention health zones than in comparison health zones (64% vs. 49%), a pattern that was observed in all sociodemographic subgroups examined except FTMs residing in medium wealth households, those with less educated parents, and those using LARCs.

### Treatment effects

After adjusting for baseline characteristics, the intervention group had a significantly higher probability of receiving FP counseling from a community-based health worker (ATE = 0.530, 95% CI = 0.480, 0.580), obtaining the current contraceptive method from a community-based health worker (ATE = 0.291, 95% CI = 0.241, 0.341), informed choice (ATE = 0.135, 95% CI = 0.067, 0.204), and of currently using implants vs. other modern methods (ATE = 0.159, 95% CI = 0.095, 0.224) than the comparison group (see Table 3). The effect of Momentum on receiving FP counseling and obtaining the current contraceptive method from a community-based health worker was significant in all socioeconomic groups examined. However, FTMs residing in medium wealth and the wealthiest households and those using LARCS did not have a significantly higher probability of informed choice in the intervention than in the comparison health zones. Regarding the probability of currently using implants vs. other modern methods, intervention efficacy was detected in all socioeconomic groups except FTMs residing in medium-wealth households. Momentum did not have any effect on use of injectables vs. other methods of contraception (see Table 2). Supplementary Table S3 shows that Momentum had a significant effect on FTMs' probability of reporting that they were very satisfied with their current contraceptive method in all sociodemographic subgroups except LARC users. Momentum had no effect on the probability of reporting that the current contraceptive method was chosen alone or jointly with the husband/male partner.

**Table 3:** Average treatment effects (ATEs) for the impact of Momentum on selected family planning outcomes, by socioeconomic characteristics, first-time mothers age 15–24 who were current modern contraceptive users, Kinshasa.

	Receiving FP Counseling from a CHW			Obtaining the Current Modern Contraceptive Method from a CHW			Informed Choice (Method Information Index)			Currently Using Implant Versus Other Modern Method			Currently Using Injectable Versus Other Modern Method		
Characteristic	ATE	95% CI		ATE	95% CI		ATE	95% CI		ATE	95% CI		ATE	95% CI	*N*
**Age group**															
15–19	0.508	(0.437, 0.580)	***	0.262	(0.193, 0.332)	***	0.124	(0.023, 0.225)	*	0.150	(0.052, 0.247)	**	−0.010	(−0.092, 0.072)	362
20–24	0.546	(0.474, 0.618)	***	0.312	(0.240, 0.385)	***	0.138	(0.044, 0.233)	**	0.175	(0.089, 0.260)	***	−0.018	(−0.097, 0.061)	399
**Marital status**															
Ever married/Formally engaged	0.527	(0.466, 0.588)	***	0.291	(0.230, 0.353)	***	0.143	(0.062, 0.225)	**	0.170	(0.096, 0.244)	***	−0.002	(−0.072, 0.068)	544
Never married	–	–	–	0.289	(0.202, 0.376)	***	0.134	(0.012, 0.257)	*	0.146	(0.021, 0.272)	*	−0.017	(−0.104, 0.071)	217
**Household wealth**															
Low	0.494	(0.406, 0.581)	***	0.318	(0.237, 0.399)	***	0.217	(0.103, 0.331)	***	0.201	(0.092, 0.310)	***	−0.016	(−0.119, 0.086)	252
Medium	0.526	(0.443, 0.609)	***	0.269	(0.185, 0.353)	***	0.105	(−0.019, 0.229)		0.115	(−0.002, 0.231)		−0.004	(−0.102, 0.093)	260
High	0.560	(0.468, 0.651)	***	0.290	(0.196, 0.384)	***	0.085	(−0.032, 0.201)		0.166	(0.056, 0.276)	**	0.009	(−0.079, 0.097)	249
**Method type**															
Short-term method	0.496	(0.431, 0.562)	***	0.196	(0.137, 0.254)	***	0.103	(0.030, 0.176)	**						517
LARC	0.583	(0.505, 0.660)	***	0.446	(0.365, 0.527)	***	−0.033	(−0.157, 0.091)							244
Total	0.530	(0.480, 0.580)	***	0.291	(0.241, 0.341)	***	0.135	(0.067, 0.204)	***	0.159	(0.095, 0.224)	***	−0.006	(−0.061, 0.050)	761

Source: Momentum 2018 Baseline Survey and 2020 Endline Survey.

ATEs are derived from an intention-to-treat analysis based on treatment effects models with inverse probability weighting. For each sociodemographic subgroup, the treatment model controls for the following baseline characteristics: single years of age, number of years of schooling, Bakongo ethnicity, parents’ education, and weekly TV exposure. The treatment model for the overall sample includes additional controls for marital status and household wealth.

- Variance matrix highly singular.

CHW, community-based health worker.

*** *p* < 0.001.

** *p* < 0.01.

* *p* < 0.05.

Tables 4, 5 examine the average effect of Momentum on the group of current users of modern contraceptives that received the project's interventions. There were four Momentum exposure variables: level of exposure, number of home visits, number of group education sessions, and total number of contacts with the project. There was strong evidence for dose-response effects of project exposure on receipt of contraceptive counseling and obtaining the current contraceptive method from a community-based health worker. For informed choice, the estimated ATET of going from no home visits to 1–3 home visits was 0.120 and the estimated ATETs of going from no home visits to 4–6 home visits and 7 or more home visits were 0.142 and 0.430, respectively. These ATETs were statistically significant. Regarding the total number of project contacts, the estimated probability of informed choice increased steadily from −0.056 for 1–3 project contacts to 0.369 for 4–6 contacts, and then declined to 0.343 for 10 or more contacts.

**Table 4:** Average treatment effects on the treated (ATET), 95% confidence intervals and *p*-values for selected outcomes by level of exposure to Momentum interventions, first-time mothers age 15–24 who were currently using a modern method, Kinshasa.

	Received FP Counseling from a CHW			Obtained the Current Contraceptive Method from a CHW			Informed Choice (Method Information Index)			
Exposure to Momentum	ATET	95% CI	Sig.	ATET	95% CI	Sig.	ATET	95% CI	Sig.	*N*
**Type of exposure**										
None (base category)										387
Partial vs. none	0.372	(0.283, 0.461)	***	0.118	(0.048, 0.190)	**	−0.044	(−0.138, 0.050)		118
Full vs. none	0.668	(0.606, 0.730)	***	0.386	(0.318, 0.453)	***	0.226	(0.143, 0.308)	***	256
**No. of home visits**										
0 (base category)										408
1–3	0.422	(0.329, 0.515)	***	0.263	(0.175, 0.352)	***	0.120	(0.018, 0.223)	*	111
4–6	0.604	(0.512, 0.694)	***	0.271	(0.180, 0.362)	***	0.142	(0.034, 0.250)	*	142
7+	0.802	(0.726, 0.877)	***	0.429	(0.325, 0.533)	***	0.430	(0.332, 0.529)	***	100
**No. of group education sessions**										
0 (base category)										484
1–2	0.477	(0.386, 0.568)	***	0.263	(0.177, 0.349)	***	0.167	(0.070, 0.262)	**	125
3–4	0.545	(0.422, 0.668)	***	0.373	(0.247, 0.499)	***	0.323	(0.204, 0.442)	***	76
5+	0.646	(0.542, 0.750)	***	0.446	(0.323, 0.568)	***	0.273	(0.147, 0.399)	***	75
**No. of times exposed to Momentum**										
0 (**base** category)										387
1–3	0.262	(0.156, 0.369)	***	0.106	(0.018, 0.194)	*	−0.056	(−0.170, 0.059)		70
4–6	0.507	(0.397, 0.616)	***	0.241	(0.137, 0.344)	***	0.038	(−0.079, 0.153)		112
7–9	0.713	(0.600, 0.826)	***	0.354	(0.223, 0.485)	***	0.369	(0.243, 0.495)	***	80
10+	0.761	(0.678, 0.844)	***	0.451	(0.345, 0.556)	***	0.343	(0.235, 0.451)	***	112

Source: Momentum 2020 Endline Survey.

ATETs are derived from an intention-to-treat analysis based on multivalued treatment effects models with inverse probability weighting. For each sociodemographic subgroup, the treatment model controls for the following baseline characteristics: single years of age, number of years of schooling, Bakongo ethnicity, parents’ education, weekly TV exposure, marital status, and household wealth.

CHW, community-based health worker.

*** *p* < 0.001.

** *p* < 0.01.

* *p* < 0.05.

**Table 5:** Average treatment effects on the treated, 95% confidence intervals and *p*-values for current use of injectables vs. other methods and current use of implants vs. other methods by level of exposure to Momentum interventions, first-time mothers age 15–24 who were currently using a modern method, Kinshasa.

	Currently Using Implant Versus Other Modern Method			Currently Using Injectable Versus Other Modern Method			
Exposure to Momentum	ATET	95% CI	Sig.	ATET	95% CI	Sig.	*N*
**Type of exposure**							
None (base category)							387
Partial vs. none	0.029	(−0.061, 0.120)		0.066	(−0.023, 0.156)		118
Full vs. none	0.238	(0.159, 0.317)	***	−0.060	(−0.121, 0.002)		256
**No. of home visits**							
0 (base category)							408
1–3	0.117	(0.015, 0.219)	*	0.003	(−0.083, 0.088)		111
4–6	0.193	(0.090, 0.295)	***	−0.028	(−0.105, 0.050)		142
7+	0.264	(0.155, 0.372)	***	−0.048	(−0.132, 0.037)		100
**No. of home visits (a)**							
0 (base category)							484
1–2	0.162	(0.017, 0.306)	*	−0.055	(−0.202, 0.091)		125
3–4	0.203	(0.058, 0.348)	**	−0.071	(−0.217, 0.074)		76
5+	0.259	(0.097, 0.420)	***	−0.062	(−0.228, 0.104)		75
**No. of group education sessions**							
0 (base category)							484
1–2	0.193	(0.095, 0.290)	***	−0.074	(−0.145, −0.005)		125
3–4	0.187	(0.062, 0.314)	**	−0.028	(−0.136, 0.080)		76
5+	0.254	(0.132, 0.376)	***	−0.027	(−0.133, 0.080)		75
**No. of group education sessions (b)**							
0 (base category)							484
1–2	−0.344	(−0.719, 0.032)		0.048	(−0.148, 0.243)		125
3–4	−0.355	(−0.749, 0.038)		0.085	(−0.126, 0.296)		76
5+	−0.369	(−0.755, 0.172)		0.164	(−0.056, 0.385)		75
**No. of times exposed to Momentum**							
0 (base category)							387
1–3	0.021	(−0.094, 0.136)		0.062	(−0.047, 0.170)		70
4–6	0.112	(−0.004, 0.228)		−0.034	(−0.116, 0.049)		112
7–9	0.271	(0.135, 0.408)	***	0.041	(−0.069, 0.151)		80
10+	0.227	(0.115, 0.339)	***	−0.043	(−0.129, 0.043)		112

Source: Momentum 2020 Endline Survey. (a) Controls for exposure to group education sessions (b) Controls for the total number of home visits

ATETs are derived from an intention-to-treat analysis based on multivalued treatment effects models with inverse probability weighting. For each sociodemographic subgroup, the treatment model controls for single years of age, number of years of schooling, Bakongo ethnicity, parents’ education, weekly TV exposure, marital status, and household wealth.

*** *p* < 0.001.

** *p* < 0.01.

* *p* < 0.05.

Regarding the other outcomes, there was strong evidence for dose-response effects of type of exposure and number of home visits on the probability of using implants vs. other modern methods. A significant increase in implant use was obtained after a total of 7–9 project contacts. Having 10 or more contacts did not significantly increase the probability of implant use relative to having 7–9 contacts (see Table 5). We did not detect a significant positive project effect on current use of injectables vs. other modern methods. As shown in Supplementary Table S4, Momentum had a positive effect on the probability that the FTM chose her current method either alone or jointly with her husband/male partner among those who participated in 5 or more group education sessions vs. none (ATET = 0.090; 95% CI = 0.002, 0.179; *p* = 0.045) and among FTMs with 7–9 project contacts vs. none (ATET = 0.093; 95% CI = 0.007, 0.180; *p* = 0.035).

### Predictors of LARC use

The results of multivariable regression models assessing the associations between explanatory variables and the choice of LARC vs. short-term methods are shown in Table 6. The analysis was stratified by age group. The first model shows that among teenage FTMs who were currently using a modern method of contraception, full vs. no exposure to Momentum interventions was associated with significantly higher odds of choosing LARCs (aOR = 2.426; 95% CI = 1.385, 4.248) as was exposure to counseling on both birth spacing and FP vs. no receipt of counseling on either topic during the prenatal period (aOR = 1.835; 95% CI = 1.061, 3.175). The model also shows that the FTM's perceived ability to ask her husband/partner to use a condom was negatively associated with the odds of LARC use among teenage FTMs.

**Table 6:** Adjusted odds ratios and 95% confidence intervals from multivariable regression model of choice of long-acting reversible contraception over short-term methods, first-time mothers age 15–24 who were currently using modern contraceptives by age group, Kinshasa.

	Age 15–19			Age 20–24			Total		
Independent Variable	AOR		95% CI	AOR		95% CI	AOR		95% CI
**Level of exposure to Momentum**									
None	–			–			–		
Partial	1.713		(0.877, 3.345)	0.934		(0.378, 2.306)	1.429		(0.852, 2.397)
Full	2.426	**	(1.385, 4.248)	3.661	***	(2.151, 6.234)	3.056	***	(2.086, 4.475)
**Exposure to counseling about birth spacing and FP**									
Neither	–			–			–		
Either birth spacing or FP	0.949		(0.403, 2.235)	1.089		(0.533, 2.227)	1.050		(0.613, 1.799)
Both birth spacing and FP	1.835	*	(1.061, 3.175)	0.891		(0.508, 1.562)	1.288		(0.874, 1.899)
FTM's years of schooling	0.948		(0.857, 1.048)	0.918		(0.830, 1.014)	0.933		(0.870, 1.000)
**Marital status**									
Ever married/formally engaged	–			–			–		
Never married	1.271		(0.743, 2.174)	1.149		(0.616, 2.144)	1.185		(0.794, 1.770)
**Ethnicity**									
Non-Bakongo	–			–			–		
Bakongo	1.033		(0.610, 1.747)	1.120		(0.663, 1.894)	1.148		(0.798, 1.652)
**Worked in the past 12 months**									
No	–			–			–		
Yes	0.920		(0.467, 1.815)	0.589		(0.330, 1.049)	0.671		(0.435, 1.035)
**Heard of both IUD and implant**									
No	–			–			–		
Yes	0.764		(0.463, 1.260)	2.038	**	(1.196, 3.472)	2.079	**	(1.238, 3.490)
**Household wealth**									
Low	–			–			–		
Medium	1.058		(0.587, 1.908)	1.154		(0.610, 2.183)	1.128		(0.735, 1.731)
High	0.763		(0.395, 1.471)	1.341		(0.695, 2.589)	1.075		(0.681, 1.696)
**Perceived ability to say no to sex**									
No	–			–			–		
Yes	1.292		(0.662, 2.521)	1.020		(0.557, 1.868)	1.162		(0.745, 1.812)
**Perceived ability to ask male partner to use a condom**									
No	–			–			–		
Yes	0.532	*	(0.286, 0.990)	0.400	**	(0.223, 0.718)	0.466	***	(0.307, 0.709)
**Unintended pregnancy**									
No	–			–			–		
Yes	2.013		(0.692, 5.853)	1.150		(0.635, 2.082)	1.269		(0.768, 2.098)
**Age 15–19**									
No							–		
Yes							1.823		(1.039, 3.200)
*Interaction Term*									
Age 15–19 × Heard of both IUD and implant							0.364	**	(0.178, 0.745)
Constant	0.327		(0.072, 1.495)	0.626		(0.145, 2.696)	0.651		(0.185, 2.296)
Log likelihood	−189.412			−189.424			−383.362		
*N*	323			376			699		

Source: Momentum 2018 Baseline Survey and 2020 Endline Survey.

*** *p* < 0.001.

** *p* < 0.01.

* *p* < 0.05.

Similar associations of level of exposure to Momentum and perceived ability to ask the husband/partner to use a condom with the odds of choosing LARCs were observed among older FTMs, as shown in second model. However, among older FTMs, LARC awareness (measured by ever hearing of both IUDs and implants) was a positive predictor of current use of LARCs (aOR = 2.038; 95% CI = 1.196, 3.472). A test of the interaction between age 15–19 and LARC awareness in the total sample of modern contraceptive users revealed that the interaction was statistically significant (aOR = 0.364; 95% CI = 0.178, 0.745; *p* = 0.006).

## Discussion

Expanding contraceptive options for young women is important for ensuring they have access to contraceptives and for meeting their needs for delaying, spacing, or limiting pregnancy. The results of our analysis suggested that overall, Momentum had significant effects on (a) receipt of FP counseling; (b) community-based contraceptive provision; (c) informed choice; (d) current use of LARCs; and (e) method satisfaction. Significant dose-response effects were detected on these outcomes for the level of exposure to Momentum interventions (none, partial, and full) and the number of home visits, with the greatest effect occurring among FTMs who were exposed to both group education sessions and home visits. All social groups examined were significantly likely to experience the effects of the Momentum interventions on FP counseling and community-based contraceptive provision, Momentum was more successful in ensuring informed choice among disadvantaged FTMs living in the poorest households than among FTMs living in medium wealth or the wealthiest households. Momentum had no effect on the use of injectables vs. other modern methods and on FTMs' participation in decision making about the current contraceptive method, except among LARC users for whom Momentum's effect was not in the expected direction. Those findings suggested that the probability of free choice was significantly greater among LARC users in comparison health zones than among those in intervention health zones.

Overall, our task-shifting approach of utilizing community-based workers to expand access to FP services and contraceptive choice has been found to be effective in other studies. These studies have used study designs that do not enable one to determine project impact or the plausibility thereof. Regardless, they have noted that there is higher comparative uptake of LARC by women attending community-based FP services (mobile outreach and special FP days) than women attending fixed health facilities (9). One study in the Republic of Niger found that married women who were counseled by a CHW had twice the odds of using a modern method of contraception compared to married women who were not (22). Regarding differences by household wealth in Momentum's effect on informed choice, we do not know if FTMs' socioeconomic status shaped how they were counseled by Momentum nursing students and whether the counseling approach facilitated FTMs' recall of counseling content. In the DRC, the use of medical/nursing students for community-based distribution of contraceptives, including both DMPA-SC and Implanon NXT, increased contraceptive access and uptake (23) and this approach has been institutionalized by the 6ème Direction of the Ministry of Health, which is responsible for the network of nursing schools throughout the DRC (24).

In this study, several factors predicted the choice of LARCs over short-term methods. Full exposure to Momentum interventions and knowledge of LARCs (implant and IUD) contributed to increased LARC use. Among younger FTMs, knowledge of LARC was not associated with LARC use, supporting previous research findings that increasing age was associated with higher LARC use (25–27) and informed choice (28). However, these findings were contrary to those of a study of 26 sub-Saharan African countries which found that older sexually active women were less likely to use LARCs (29). The results of our study also suggested that empowered women (those able to ask a male partner to use a condom) had lower odds of using LARCs compared to women who were not similarly empowered. This observed negative association does not align with findings from studies conducted in other countries showing that that empowered women have higher odds of contraceptive use (30, 31).

Our data showed that seven in ten FTMs in comparison health zones obtained their current contraceptive method from the private sector. To better understand our findings, it would be important to compare counseling content between Momentum nursing students and health care providers in both public and private fixed health facilities in comparison health zones. It is also unclear as to the extent to which waiting time and client volume may have influenced counseling content in fixed facilities in comparison health zones. These challenges were less likely to have been experienced during home visits and group education sessions in intervention health zones. It is also possible that fewer FTMs in the intervention health zones may have decided on a method at the time of the initial postpartum home visit and may have received more comprehensive counseling from Momentum nursing students. In comparison health zones, FTMs may have experienced less comprehensive counseling if they had already decided on the method they wanted to use and had obtained that method from a fixed facility. Differences may have also occurred if contraceptive provision and counseling were done by different providers in fixed facilities. The assignment of dedicated nursing students to FTMs in intervention health zones and home visits by the same nursing students to a given FTM for about 16 months may have ensured that contraceptive counseling and service provision were more tailored to the needs of the individual FTM client.

### Study limitations

The main limitation of the study was that our results could not be generalized to FTMs in Kinshasa as our sample was purposive. Recall bias and courtesy bias were additional limitations and may have affected the reliability of our results, especially regarding our measurement of the effect of Momentum on method satisfaction. Additionally, our analysis did not consider health worker-related factors which have been found to influence contraceptive choice. A recent systematic review of studies conducted in 27 countries suggested that the provision of counseling was influenced by health workers' values and preferences as well as by their misconceptions and biases (32). Thus, the nursing students' views may have influenced their provision of FP counseling. Finally, as FTMs were recruited when they were six months pregnant, it was impossible to observe contraceptive use at baseline before the Momentum interventions were implemented. While we could not randomize receipt of the Momentum interventions, which would have enabled us to establish causality, our treatment effects models have allowed us to estimate project efficacy using observational data.

## Conclusions

The present study suggests that deploying nursing students to provide family planning services through home visits is an important way to increase adolescent and young FTMs' access to contraceptive counseling and methods, including implants, and informed choice. It will be important to understand why contraceptive choice was more difficult to obtain in comparison health zones and to examine ways of ensuring comprehensive FP counseling. The project had negligible effects on empowering FTMs to make decisions about the current contraceptive method and future programs need to address this issue. Although the nursing student model for administering contraception, including injectables and implants has been institutionalized in the nursing curriculum in the DRC (23, 33), the scale-up of Momentum approach would require that attention be given to resupply, correct disposal of medical waste, and the establishment of referral linkages between nursing schools and fixed health facilities for management of side effects and LARC removal.

## Data Availability

The raw data supporting the conclusions of this article will be made available by the authors, without undue reservation.
