# Supplementary material for: Expanding contraceptive choice among first-time mothers age 15–24 in Kinshasa: The Momentum pilot project
Source: Front Glob Womens Health. 2023 Feb 13;4:1087009. doi: 10.3389/fgwh.2023.1087009 (PMC9969108; doi:10.3389/fgwh.2023.1087009)
Supplement: Supplementary file 1 [file Datasheet1.pdf]

Supplementary Material

Supplementary Table 1 Percent distribution of the sample with missing and non-missing values on variables included in the analysis of LARC use, FTMs age 15-24 who were currently using a modern method of contraception, Kinshasa, DRC

		Non-missing			All FTMs currently using Modern Contraception
	Missing	Age 15-19	Age 20-24	Total	
**Exposure to Momentum**					
None	43.5	48.6	54.0	51.5	50.9
Partial	12.9	19.2	12.8	15.7	15.5
Full	43.5	32.2	33.2	32.8	33.6
**Exposed to BS and/or FP counseling in 6 months preceding baseline survey**					
Neither	50.8	57.3	46.3	51.4	51.3
BS or FP	11.9	11.1	17.0	14.3	14.1
Both (BS & FP)	37.3	31.6	36.7	34.3	34.6
**Marital status *****					
Ever married/formally engaged	51.6	66.9	78.7	73.2	71.5
Never married	48.4	33.1	21.3	26.8	28.5
**Ethnicity**					
Non-Bakongo	64.5	62.8	62.0	62.4	62.5
Bakongo	35.5	37.2	38.0	37.6	37.5
**Worked in the past 12 months**					
No	64.4	81.7	68.9	74.8	74.0
Yes	35.6	18.3	31.1	25.2	26.0
**Ever heard of LARCs**					
No	33.9	48.3	42.0	44.9	44.0
Yes	66.1	51.7	58.0	55.1	56.0
**Household wealth**					
Low	41.9	34.7	30.3	32.3	33.1
Medium	29.0	34.7	34.6	34.6	34.2
High	29.0	30.7	35.1	33.0	32.7
**Unintended pregnancy ***					
No	6.8	21.4	22.3	17.2	16.4
Yes	93.2	78.6	77.7	82.8	83.6
Total	100.0	100.0	100.0	100.0	100.0
**Number of FTMs**	**62**	**323**	**376**	**699**	**761**

Source: Momentum 2018 Baseline Survey and 2020 Endline Survey
BS Birth spacing
FP Family planning
FTM First-time mother
LARC Long-acting reversible contraception
*** $p < 0.001$; * $p < 0.05$ (significance of differences between missing and total non-missing cases)

Supplementary Table 2 Percentage of modern contraceptive users age 15-24 who chose the current contraceptive method alone or jointly with their male partner, and who were very satisfied with the current method, by baseline characteristics and health zone, first-time mothers, Kinshasa

	Chose Method Alone or Jointly with Male Partner			Very Satisfied with Current Method		
**Characteristics**	**Comparison**	**Intervention**	**Sig.**	**Comparison**	**Intervention**	**Sig.**
**Highest level of schooling**						
None/prim/sec. inc.	81.4	76.2		46.4	63.5	***
Sec. complete/higher	78.9	81.9		52.0	63.7	*
**Marital status**						
Ever married/formally engaged	79.4	79.7		48.0	60.8	**
Never married	82.7	75.6		51.0	70.6	*
**Household wealth**						
Low	77.9	76.3		43.4	66.2	***
Medium	84.4	78.8		53.2	62.3	
High	79.0	80.8		50.0	62.4	*
**Worked last year**						
No	80.2	76.1		50.2	63.0	**
Yes	80.6	83.5		46.8	64.7	**
**Watched TV weekly**						
No	80.2	74.0		49.6	68.0	**
Yes	80.4	81.1		48.4	61.1	**
**Both parents have secondary/higher education**						
No	75.7	74.1		50.0	58.8	
Yes	81.5	79.7		48.6	64.8	***
**Method type**						
SAC	78.0	77.5		44.4	59.8	***
LARC	88.5	80.1		64.1	69.3	
Total	80.3	78.6		48.8	63.6	***

Source: Momentum 2018 Baseline Survey and 2020 Endline Survey
*** p<.001; ** p< 0.01; * p < 0.05. P-values pertain to differences between health zones.

Supplementary Table 3 Average treatment effects (ATEs) of Momentum on the probability of choosing the current contraceptive method alone or jointly with the male partner and of being very satisfied with the current method, by selected socioeconomic characteristics, first-time mothers age 15-24 who were currently using modern contraceptives, Kinshasa

	Chose Current Contraceptive Method Alone or Jointly with Male Partner			Very Satisfied with Current Contraceptive Method			
**Characteristic**	**ATE**	**95% CI**	**P-value**	**ATE**	**95% CI**	**P-value**	**N**
**Age group**							
15-19	-0.032	(-0.121, 0.057)	0.477	0.174	(0.071, 0.277)	<0.001	362
20-24	-0.006	(-0.079, 0.068)	0.882	0.122	(0.024, 0.219)	0.015	399
**Marital status**							
Ever married/formally engaged	0.007	(-0.061, 0.074)	0.846	0.130	(0.047, 0.214)	0.002	544
Never married	-0.073	(-0.178, 0.032)	0.171	0.201	(0.070, 0.332)	0.003	217
**Household wealth**							
Low	-0.024	(-0.127, 0.078)	0.643	0.232	(0.116, 0.349)	<0.001	252
Medium	-0.032	(-0.130, 0.066)	0.523	0.128	(0.005, 0.251)	0.041	260
High	0.009	(-0.092, 0.110)	0.862	0.124	(0.001, 0.247)	0.049	249
**Method type**							
SAC	-0.002	(-0.074, 0.068)	0.937	0.155	(0.070, 0.239)	<0.001	517
LARC	-0.093	(-0.182, -0.003)	0.043	0.051	(-0.077, 0.179)	0.436	244
Total	-0.019	(-0.076, 0.038)	0.521	0.151	(0.081, 0.220)	<0.001	761

Source: Momentum 2018 Baseline Survey and 2020 Endline Survey

ATEs are derived from an intent-to-treat analysis based on treatment effects models with inverse probability weighting. For each sociodemographic subgroup, the treatment model controls for the following baseline characteristics: single years of age, number of years of schooling, Bakongo ethnicity, parents' education, and weekly TV exposure. The treatment model for the overall sample includes additional controls for marital status and household wealth.

Supplementary Table 4 Average treatment effects on the treated, (ATET), 95% confidence intervals, and p-values for decision making about the current method and method satisfaction by level of exposure to Momentum interventions, first-time mothers age 15-24 who were currently using a modern method, Kinshasa

	Chose Current Contraceptive Method Alone or Jointly with Male Partner			Very Satisfied with Current Contraceptive Method			
**Exposure to Momentum**	**ATET**	**95% CI**	**P-value**	**ATET**	**95% CI**	**P-value**	**N**
**Type of exposure**							
None (base category)							387
Partial versus none	-0.055	(-0.147, 0.036)	0.238	0.054	(-0.051, 0.159)	0.311	118
Full versus none	-0.005	(-0.074, 0.063)	0.881	0.216	(0.137, 0.295)	<0.001	256
**Number of home visits**							
0 (base category)							408
1-3	-0.076	(-0.168, 0.015)	0.103	0.022	(-0.085, 0.129)	0.687	111
4-6	0.013	(-0.068, 0.096)	0.740	0.238	(0.144, 0.332)	<0.001	142
7+	0.038	(-0.049, 0.124)	0.397	0.254	(0.151, 0.356)	<0.001	100
**No. of group education sessions**							
0 (base category)							484
1-2	-0.051	(-0.136, 0.034)	0.244	0.106	(0.009, 0.203)	0.031	125
3-4	-0.008	(-0.122, 0.104)	0.878	0.154	(0.034, 0.273)	0.012	76
5+	0.090	(0.002, 0.179)	0.045	0.356	(0.264, 0.447)	<0.001	75
**No of times exposed to Momentum**							
0 (base category)							387
1-3	-0.104	(-0.222, 0.015)	0.086	-0.053	(-0.181, 0.075)	0.418	70
4-6	-0.043	(-0.152, 0.067)	0.443	0.235	(0.130, 0.339)	<0.001	112
7-9	0.093	(0.007, 0.180)	0.035	0.136	(-0.003, 0.276)	0.056	80
10+	0.012	(-0.083, 0.107)	0.805	0.314	(0.215, 0.412)	<0.001	112

Source: Momentum 2020 Endline Survey

ATETs are derived from an intent-to-treat analysis based on multivalued treatment effects models with inverse probability weighting. For each sociodemographic subgroup, the treatment model controls for the following baseline characteristics: single years of age, number of years of schooling, Bakongo ethnicity, parents' education, weekly TV exposure, marital status and household wealth.
